# The impact of environmental stimuli on the psychological and behavioral compliance of international construction employees

**DOI:** 10.3389/fpsyg.2024.1395400

**Published:** 2024-06-11

**Authors:** Tengyuan Chang, Yi Wu, Xiaopeng Deng, Xianru Wang, Yangzhi Yan

**Affiliations:** ^1^Institute of Human Rights, Law School, Southeast University, Nanjing, China; ^2^China-Pakistan Belt and Road Joint Laboratory on Smart Disaster Prevention of Major Infrastructures, Southeast University, Nanjing, China

**Keywords:** compliance management, international engineering, corruption, SOR model, organizational behavior

## Abstract

**Introduction:**

This study explores the overlooked psychological and behavioral dynamics of employees in compliance management, applying the Stimulus-Organism-Response (SOR) framework to assess environmental stimuli’s impact on employees in international construction projects.

**Methods:**

A scenario-based survey involving 270 international construction employees was analyzed using Partial Least Squares Structural Equation Modeling (PLS-SEM) and Necessary Condition Analysis (NCA), focusing on the relationship between environmental stimuli and compliance intentions.

**Results:**

Findings categorize environmental influences on compliance into internal and external organizational dimensions, highlighting the significant impact of internal factors on compliance intentions. Key determinants identified for high compliance intention include individual traits and organizational climate, while project pressures, rules and regulations, and cultural differences show variable influence.

**Conclusion:**

This study enhances the understanding of the psychological factors driving non-compliant behaviors and introduces a binary micro-ecological approach to compliance management, effectively integrating individual and project organizational elements. In contrast to traditional corporate governance approaches, this strategy emphasizes the role of project organizational micro-ecology in the management of international construction projects. The strategy aims to improve compliance management among international contractors by influencing the psychological and behavioral compliance of frontline employees.

## 1 Introduction

In the globalized context, international construction projects are vital to cross-border infrastructure, engineering management, and technical exchange, bridging economic and cultural divides. These complex projects, extending beyond technical and managerial aspects, are situated within diverse legal, cultural, and regulatory contexts (Chan and Tse, 2003). This complexity underscores the importance of sophisticated compliance management strategies for international contractors, reflecting the dynamic challenges of global construction practices (Gao et al., 2023).

Compliance management, essential in international construction, involves aligning operations with legal, industry, and ethical standards (Zhao et al., 2012). Its importance is amplified in multinational projects involving various stakeholders and legal jurisdictions. Non-compliance can result in reputational damage, financial penalties, and even criminal charges (Rabbiosi and Santangelo, 2019; Bahoo et al., 2020; Wang et al., 2021). For instance, Specifically, in World Bank-financed projects, breaches such as fraud, collusion, corruption, or coercion can lead to debarment and cross-debarment from other development banks, underscoring the need for rigorous compliance adherence (Soreide et al., 2015).

Despite the acknowledged importance of compliance management in international construction, its implementation faces significant challenges. Studies indicate prevalent non-compliance in this sector, Such as bribery, corruption, conflict of interest and nepotism (Monteiro et al., 2022; Soni et al., 2024), often due to employees’ limited understanding of compliance standards. This superficial grasp leads to ineffective risk management (Okoye et al., 2016; Oluseye et al., 2024). Furthermore, many contractors adopt top-down compliance strategies, not fully addressing the unique demands of international projects or the specific needs of field employees, which impairs effective global risk mitigation.

In corporate governance, the efficacy of compliance management in curbing non-compliant worker behaviors is critical (Soltes, 2017). Worker compliance behavior is deeply rooted in their mindset and intentions (Furnham, 2012). The complexity and diversity of international construction projects require flexibility and precision in worker decision-making (Ofori, 2015). Environmental factors such as legal constraints, organizational climate, and project pressures significantly influence employees’ perception and emotional response to compliance (Gendolla, 2000; Al Jaffri Saad and Haniffa, 2014). Misconceptions or disagreements among employees about compliance can lead to non-compliant behaviors (Syahputra et al., 2022), emphasizing the need to understand the environmental triggers of psychological processes leading to compliance.

Current research in international construction compliance focuses on establishing compliance plans (Luo et al., 2022), assessing compliance (Yu et al., 2014), and identifying factors driving non-compliance like corruption (Shan et al., 2017). These studies emphasize macro-level and organizational aspects, such as regulatory and industry environments (Ben Khaled and Gond, 2020; Liu et al., 2024), yet underplay individual-level influences. This oversight points to the need for deeper examination of how individual perceptions within specific contexts shape compliance behaviors.

In research related to individual compliance, scholars have identified several key factors influencing compliance behavior in construction projects. Studies indicate that compliance among junior employees in indigenous construction companies is generally poor, often due to inadequate government supervision and lax law enforcement (Ebekozien, 2022). Additionally, the impact of environmental factors on individual compliance cannot be ignored. External environments, such as the completeness and effectiveness of the host country’s laws, profoundly affect employees’ perception of compliance risk (Liu et al., 2024), while internal environments, such as transformational leadership, also play a significant role (Shen et al., 2017). The characteristics of employees themselves are crucial as well. Individual traits such as situational awareness and emotional intelligence have a notable impact on safety compliance behavior, with the ability to regulate emotions being particularly important (Yan et al., 2023). Differences in cognitive levels also lead to variations in individuals’ adherence to safety norms (Ijaola et al., 2021). Overall, while existing research has made progress in understanding compliance at the macro and organizational levels, there is a need for greater focus on individual-level influences to comprehensively understand and improve compliance behavior in construction projects.

The Stimulus-Organism-Response (SOR) framework is pivotal for understanding the interaction between environmental factors and individual psychological processes in compliance behavior. This model posits environmental stimuli as influencing behavior via psychological processes (Jacoby, 2002; Laato et al., 2020). In international construction, recognizing how environmental factors affect worker psychology and behavior is crucial for designing effective compliance strategies. This approach contributes significantly to both theory and practice in managing compliance in complex project environments.

This study innovatively applies the SOR model to compliance management in international construction, identifying environmental factors influencing professional psychology and behavior. It suggests targeted strategies for optimizing environmental stimuli to guide compliance behavior effectively. This research enhances the theoretical understanding of psychological and behavioral processes in compliance management and provides novel strategies for international contractors, bridging theory with practical application in this dynamic field.

## 2 Theory and hypothesis

In behavioral sciences, while theories like Planned Behavior (TPB), Value-Belief-Norm (VBN) Theory, and Norm Activation Model (NAM) focus on attitudes and values, they often overlook environmental and emotional influences on behavior. Contrasting this, the SOR theory, an evolution of Thorndike’s SR (Stimulus–Response) framework, offers a comprehensive understanding by emphasizing the impact of external stimuli on psychological states and subsequent behaviors. The SOR model considers “stimulus” as external inputs, “organism” as cognitive and emotional processes, and “response” as resulting behavior, highlighting the intricate relationships between the environment and individual psychological processes (Moore, 2011; Song et al., 2022).

Traditionally used in consumer behavior research, the SOR theory describes how external stimuli evoke perceptual and emotional changes, leading to specific responses. This theory has been effectively applied to understand behavioral responses to environmental stimuli in various contexts, ranging from university students’ enrollment intentions to psychological changes during crises and public low-carbon behaviors (Nagoya et al., 2021; Pandita et al., 2021). In international construction projects, worker compliance is often directly influenced by environmental stimuli like legal and organizational factors. Thus, applying the SOR model to explore compliance behavior in this context is theoretically sound, providing a comprehensive framework that captures the complexities of international construction environments.

### 2.1 Environmental stimuli

“Environmental stimuli” in the SOR model refers to external influences processed through perception and emotional transformation, impacting individual behavior. In international construction, this encompasses both immediate environments like organizational culture and broader external factors such as industry norms and legal regulations, shaping worker behavior and attitudes (Hu et al., 2023).

Focusing on internal organizational elements, employees’ compliant behavior is shaped by “Individual Traits,” “Organizational Climate,” and “Project Pressures.” Research indicates personal capabilities, cognitive states, and work experience are key (Mitropoulos and Memarian, 2012; Humaidi and Balakrishnan, 2015; Chen et al., 2018). These traits, processed emotionally and perceptually, act as stimuli impacting behavior within the SOR framework (Moore, 2011). Organizational factors like measures (Stachowicz-Stanusch and Simha, 2013), transparency (Oyewobi et al., 2011), strategies (Wicks et al., 1999), and ethics link to non-compliance (Hansen and Beukema, 2014). These elements collectively form the “Organizational Climate,” representing employees’ shared perceptions and meanings about policies, practices, procedures, rewards, support, and expectations (Kutsch and Hall, 2010). Additionally, the project’s inherent pressures, including cost (Bowen et al., 2012), time (Tow and Loosemore, 2009), quality (Stansbury, 2005), and various other factors, serve as environmental stimuli influencing employees’ compliance decisions in international construction projects.

From the external organizational view, the stimuli can be classified into “Rules and Regulations” and “Cultural Differences “where the former guides compliance through legal policies, and the latter shapes compliance perceptions and behaviors via cultural variations. Policy formulation and legal enforcement provide essential guidance and boundaries for employees’ compliance behaviors (Sohail and Cavill, 2008). cultural differences affect not only work styles and communication patterns but also the interpretation and application of compliance standards (Yean Yng Ling et al., 2007). The impact of diverse cultural backgrounds on understanding and practicing compliance cannot be overlooked, as employees need to understand and adapt to these differences to make decisions that align with project requirements and objectives (Pheng and Leong, 2000).

### 2.2 Organism

In the SOR framework, “Organism” represents the cognitive and affective processes mediating between stimuli and responses. Attitudes, central to these processes, are predispositions influencing actions and behavior (Choi and Park, 2017). Widely acknowledged as predictors of behavioral intentions, attitudes are used as mediators across various contexts, including trust and behavior-specific attitudes (Arora et al., 2020; Tuncer, 2021). Thus, “Attitude toward Compliance” is a critical organism factor in this study.

In the SOR paradigm for consumer behavior, perceived value is a psychological assessment of cost–benefit analysis, influencing decisions (Chen and Prompanyo, 2021). Applied to compliance in international construction, this concept equates compliance to consumer behavior, shaped by cost, benefit, and reputation. Hence, “Perceived Behavior Value” is another crucial organism factor in this study, reflecting the psychological evaluation process in the context of compliance.

Interpersonal Behavior Theory (TIB) differentiates emotions from attitudes, suggesting environmental emotions manifest as positive or negative states (Triandis, 1979). Emotions, as psychological responses, are crucial in decision-making (Lewis et al., 2017). In the SOR framework, emotions, often categorized as positive or negative, play a key role as mediators (Song et al., 2022). “Emotional Positivity,” representing the positive psychological state elicited by compliance, is thus an essential organism factor in this study.

### 2.3 Response

In the SOR model, “Behavioral Intention” is a crucial concept for understanding individual behavior (Hofeditz et al., 2017). It’s commonly used as a response measure in studies, like gaging purchase intention in consumer behavior research (Tuncer, 2021). In international construction projects, “Compliance Intention” serves as a key predictor of compliant behavior, reflecting underlying motivations and likelihood of adherence to norms (Al Jaffri Saad and Haniffa, 2014). This study, therefore, incorporates “Compliance Intention” as the response variable, exploring the drivers of compliance behavior among employees in these projects.

### 2.4 Hypothesis

In international construction projects, individual traits like compliance experience and rule adherence are crucial for shaping positive compliance behaviors (Jaafar and Ajis, 2013). These traits are essential for making appropriate judgments across diverse legal and cultural landscapes, ensuring the integrity and reliability of project processes. Moreover, these individual characteristics significantly influence emotional stability and engagement in compliance-related tasks (Henle and Gross, 2014). Particularly under the pressures and challenges inherent in international construction projects, these traits contribute to maintaining a positive emotional disposition, mitigating the adverse effects of work-related stress and fostering a commitment to compliant behavior. Accordingly, this study posits the following hypotheses:

*Hypothesis 1*: Individual traits have a significant influence on attitude toward compliance.

*Hypothesis 2*: Individual traits have a significant influence on perceived behavior value.

*Hypothesis 3*: Individual traits have a significant influence on emotional positivity.

Organizational climate profoundly influences employees’ compliance behavior, with the organizational environment substantially shaping their enthusiasm for compliance (Cirka, 2000; Sillic, 2019). The culture and atmosphere of an organization significantly impact the psychological processes employees undergo when making decisions about compliance behavior (Schneider and Hall, 1972). A culture that emphasizes the importance of compliance can enhance employees’ attitudes toward compliant behavior during their work (Hu et al., 2012). Research indicates a positive correlation between organizational climate and organizational corruption (Stachowicz-Stanusch and Simha, 2013), suggesting that a compliance-oriented organizational atmosphere can reduce the allure of non-compliant behavior and heighten the value employees place on compliance. Moreover, a clear and comprehensive compliance plan provides a guideline for action, boosting employees’ positive emotional commitment and adherence to compliance amidst the complexities of international construction projects (Liu et al., 2017). Based on these insights, the study proposes the following hypotheses:

*Hypothesis 4*: Organizational climate significant influence attitude toward compliance.

*Hypothesis 5*: Organizational climate significant affect perceived behavior value.

*Hypothesis 6*: Organizational climate significant impact emotional positivity.

Project pressures, encompassing stringent timeframes, quality benchmarks, and cost control, along with intensified competition among contractors, represent a formidable aspect of international engineering endeavors (Dulaimi and Shan, 2002). In such high-stakes settings, employees may confront a dilemma between project success and adherence to compliance standards. The exigencies of project objectives can compel a compromise between efficiency and regulatory obedience. Despite a proactive stance toward compliance, the immediate pursuit of project milestones can overshadow the perceived value of compliance behaviors. Under such conditions, if compliance actions do not facilitate the attainment of project goals, the employees’ emotional positivity toward compliance could wane. Based on these considerations, the study posits the following hypotheses:

*Hypothesis 7*: Project pressures significant influence the perceived behavior value.

*Hypothesis 8*: Project pressures significant affect emotional positivity.

Clear and precise regulations and a keen understanding of cultural differences are essential for ensuring the smooth operation of international contractors’ businesses. These rules provide guidance for employees’ behavior against a backdrop of diverse cultures, while strict enforcement by regulatory bodies deepens employees’ recognition and value of compliance adherence. With explicit punitive mechanisms in place, employees more seriously consider the long-term value of compliance behaviors to avoid potential adverse outcomes (Manu et al., 2013). Moreover, the culture of the host country offers a framework of values and ethics for employees’ daily conduct, facilitating their understanding and integration into local business customs (Liu et al., 2015). However, if employees fail to adapt to cultural differences, their valuation and emotional investment in compliance behaviors may be negatively impacted, increasing compliance risks, and potentially leading to decreased efficiency and quality in compliance implementation. Therefore, this study posits the following hypotheses:

*Hypothesis 9*: Rules and regulations significant impact attitude toward compliance.

*Hypothesis 10*: Rules and regulations significant influence perceived behavior value.

*Hypothesis 11*: Cultural differences significant affect perceived behavior value.

*Hypothesis 12*: Cultural differences significant effect emotional positivity.

Attitude toward Compliance reflects a psychological inclination, considered a significant predictor of behavioral intention (Choi and Park, 2017). In international construction projects, the decision-making process of employees regarding compliance behaviors can be likened to a consumer’s purchasing process. Employees engage in compliant behaviors based on a judgment of perceived value, where the perceived benefits outweigh the costs, and perceived value is the most direct factor influencing consumer purchase behaviors (Chen and Prompanyo, 2021). Moreover, perceived value has been associated with a positive correlation with continuance intentions (Wang et al., 2020). Emotions play a crucial role in an individual’s ultimate behavior choices (Lewis et al., 2017), and research has identified a positive effect of emotions on behavior (Ibanez et al., 2017). Drawing upon these considerations, this study posits the following hypotheses:

*Hypothesis 13*: Attitude toward compliance significant influence compliance intention.

*Hypothesis 14*: Perceived behavior value significant affect compliance intention.

*Hypothesis 15*: Emotional positivity significant impact compliance intention.

**Figure 1:**
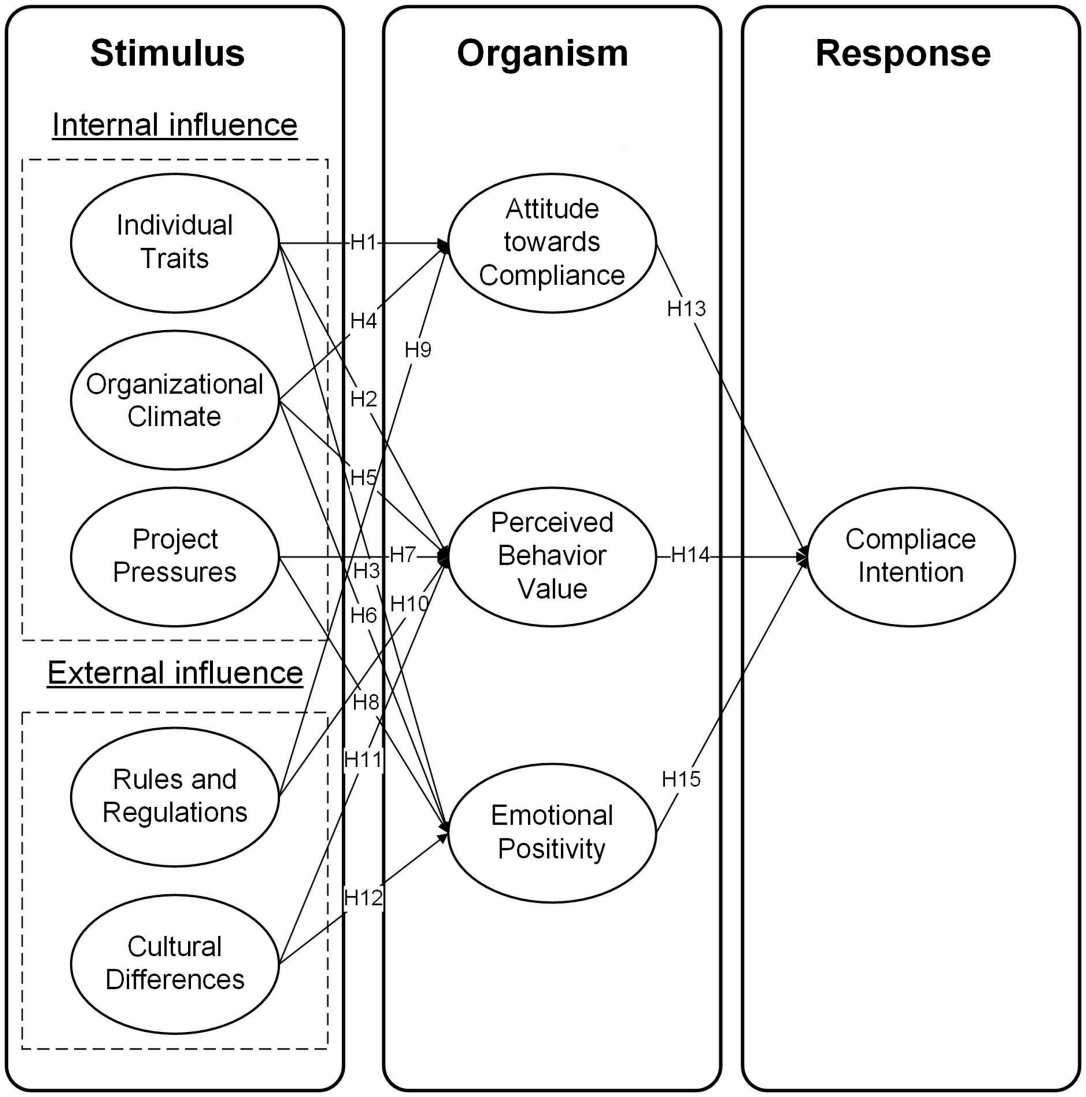
Hypothesized model.

Drawing on the preceding hypotheses, the conceptual model is depicted in Figure 1.

## 3 Methods

### 3.1 Sample and data collection

In this study, a choice-based questionnaire survey was utilized as the data Research Topic method, as the variables within the model are subjective in nature. Considering the sensitive and covert aspects of compliance issues, three measures were employed to reduce defensive responses from the respondents (Brown and Loosemore, 2015). Firstly, to alleviate any concerns or apprehensions of the participants, it was clarified that the purpose of the questionnaire was academic research, with no right or wrong answers required, but rather responses based on personal experience. Secondly, participants were assured of the survey’s complete anonymity, ensuring that no personal information or results of the survey would be disclosed. Lastly, a hypothetical scenario method was adopted, where respondents were asked to answer questions based on scenarios rather than real events, which aids in mitigating social desirability bias (Hofeditz et al., 2017; Hong and Furnell, 2022).

To ensure the scenarios were easily comprehensible to respondents, common compliance situations were initially identified from case studies. Subsequently, interviews were conducted with five employees possessing extensive experience in international construction projects, to select the most suitable scenario for the questionnaire survey. The final scenario is as follows: Imagine Tom working on an international construction project. In this project, he is responsible for ensuring that the work is completed on time and within budget, while strictly adhering to the environmental, social, and governance (ESG) standards of the host country, and effectively preventing any corruption or fraud. Tom’s role involves collaborating with colleagues from diverse cultural backgrounds, navigating a range of complex legal and ethical issues, and managing the dual pressures of project progress and cost. In this multifaceted and challenging environment, one of the primary challenges he faces is finding a balance between complying with regulations and achieving project objectives. In this context, how would you anticipate Tom’s behavior?

The snowball sampling method was employed for data Research Topic for two main reasons. First, given the uncertainty in the number of personnel with international engineering experience, snowball sampling provides a statistically reliable sample (Oyewobi et al., 2019). Second, this method allows respondents to suggest individuals interested in participating in the survey. Considering the sensitive nature of compliance issues (Rabl and Kühlmann, 2008), this approach aids in obtaining more reliable data. Initially, 50 respondents were selected from the China Architectural Society, the alumni association of the researcher’s university, and the China International Contractors Association. They were then asked to recommend additional individuals suitable for the study. It is worth considering that the survey list originates from individuals who have been previously surveyed, and there may be similarities among them, which could potentially lead to deviations in the overall sample. At the outset of the survey, this study required surveyees to provide a list that meets the requirements while avoiding homogeneity as much as possible. Ultimately, 300 questionnaires were distributed, of which 273 were retrieved. Three were discarded due to incomplete or clearly erroneous responses, resulting in 270 valid questionnaires. Given the sensitivity surrounding compliance issues, obtaining a large sample size is somewhat unrealistic (Brown and Loosemore, 2015). The sample size for this study, while relatively small, is sufficient for an exploratory study involving a highly sensitive topic (Gunduz and Önder, 2013).

### 3.2 Characteristic of respondents

In terms of work experience, the majority of the 270 respondents had more than 5 years of relevant experience, with 42.96% having over 10 years of experience. In terms of position, 25.93% were in corporate management positions, including senior managers and department managers, while project managers and engineers working at the grassroots level of international construction projects accounted for 20.74 and 43.70% of respondents, respectively. Geographically, 85 respondents were from China, while the remaining 185 were working on international construction projects in Asia (excluding China), Africa, Europe, North America, South America, and Australia (Table 1).

**Table 1:** Demographic characteristics of respondents.

Characteristics	Items	Frequency	Percentage
		(*n* = 270)	
Work experience	<3	21	7.78
	3–5	48	17.78
	6–10	85	31.48
	11–15	67	24.81
	>15	49	18.15
Rank	Senior manager	42	15.56
	Department manager	28	10.37
	Project manager	56	20.74
	Engineer	118	43.7
	Else	26	9.63
Area	China	85	31.48
	Asia (except China)	85	31.48
	Africa	49	18.15
	Europe	14	5.19
	North America	8	2.96
	South America	11	4.07
	Australia	18	6.67

### 3.3 Measures

The measurement items for the survey were initially developed based on a comprehensive review of relevant literature and were further tailored to fit the specifically designed scenarios of international construction compliance. To ensure the survey’s validity and reliability, a pilot study was conducted with 10 experts in the field of international construction, comprising 5 executives from international construction firms and 5 academics from both domestic and international universities. The survey’s questions were refined based on feedback from this pilot study. Following this, a select group of employees with significant experience in international construction was chosen to complete the questionnaire before its official distribution, to test its content and structure. The survey was structured into three sections: (1) a brief explanation of the designed scenarios related to international construction compliance; (2) questions pertaining to personal information such as years of work experience; and (3) the evaluation of related items (Table 2) based on respondents’ experience, using a Likert five-point scale ranging from 5 (strongly agree) to 1 (strongly disagree).

**Table 2:** Scale items.

Category	Items	References
Individual traits (IT)	C1: Tom has compliance experience	Kwofie et al. (2018)
	C2: Tom actively monitors external information	Wang and Mao (2012)
	C3: Tom adheres to rules	Peng and Chan (2019)
Organizational climate (OC)	B1: Tom’s organization prioritizes compliance for growth	Weaver and Treviño (1999)
	B2: Tom’s organization routinely engages in compliance activities	Li and Liu (2022)
	B3: Tom’s organization has a dynamic, explicit, and comprehensive compliance plan	Paine (1994)
Project pressures (PP)	C1: Tom feels relaxed and manages well even under tight deadlines	Cohen et al. (1983)
	C2: Tom efficiently balances maintaining project progress with compliance requirements	Mendl (1999)
	C3: Despite budget and time constraints, Tom consistently achieves full compliance	Beautement et al. (2008)
Rules and regulations (RR)	D1: Tom clearly understands the laws and regulations of the project’s host countries	Aaltonen (2013)
	D2: Regulatory bodies strictly enforce rules	Djankov et al. (2002)
	D3: Tom can effectively apply these rules and regulations in his work	Pistor et al. (2000)
Cultural differences (CD)	E1: Tom feels comfortable communicating with colleagues from different cultural backgrounds	Hofstede (1984)
	E2: Tom adapts his work style to align with different cultural norms in the project	Earley and Ang (2003)
	E3: Tom understands how cultural differences can impact project outcomes	Thomas (2008)
Attitude toward compliance (AC)	F1: Tom believes that strict adherence to compliance is crucial for project success	Ajzen (1991)
	F2: Tom views compliance as a moral obligation in his professional role	Treviño et al. (2006)
	F3: Tom considers compliance as an integral part of project management	Weaver and Trevino (2001)
Perceived behavior value (PBV)	G1: Tom perceives that following compliance guidelines adds value to the project	Rokeach (1973)
	G2: Tom believes that compliance activities contribute to the long-term sustainability of organization	Schwartz (1992)
	G3: Tom views compliance as a means to enhance the company’s reputation and trustworthiness	Donaldson and Dunfee (1999)
Emotional positivity (EP)	H1: Tom feels confident in his ability to handle compliance-related challenges	Bandura et al. (1999)
	H2: Tom maintains a positive attitude when dealing with compliance issues	Fredrickson (2001)
	H3: Tom feels motivated when working on compliance aspects of the project	Ryan and Deci (2000)
Compliance intention (CI)	I1: Tom intends to strictly follow all compliance regulations in his work	Fishbein and Ajzen (1977)
	I2: Tom plans to actively seek additional training or information related to compliance	Ajzen (2002)
	I3: Tom is committed to advocating for compliance within his project team	Kurland (1995)

### 3.4 Data analysis

Structural Equation Modeling (SEM) was utilized in this study to elucidate and substantiate the interrelations among diverse constructs, while Necessary Condition Analysis (NCA) was applied to ascertain if environmental factors constitute indispensable conditions for the realization of Compliance Intention. The integration of SEM and NCA in data analysis allows for a comprehensive consideration of the intricacies inherent in causal relationships and the essentiality of conditions (Gao et al., 2023), thereby enriching the understanding of the role environmental factors play in the formation of compliance intention.

Due to the presence of nonnormal data and the constraint of a small sample size (Ringle et al., 2012; Le et al., 2014), the study utilized Partial Least Squares Structural Equation Modeling (PLS-SEM), with 270 samples deemed sufficient for robust analysis. Data analysis was conducted using Smart PLS 3 statistical software (Ringle et al., 2015), employing the “path” weighting scheme and setting the maximum iteration count to 300. For the purpose of bootstrapping, a bias-corrected and accelerated approach was chosen, involving 5,000 subsamples to ensure the accuracy and reliability of the results. To assess the potential impact of common method bias, Harman’s one-factor test was applied. The outcome of an unrotated exploratory factor analysis revealed that the most substantial of the 27 extracted factors accounted for only 30.13% of the total variance. This proportion, being significantly below the often-cited threshold of 50%, suggested the absence of common method bias in the study. Additionally, the application of PLS-SEM further mitigates concerns regarding response biases, a noted advantage of this methodology (Pertusa-Ortega et al., 2010). Confirmatory statistical tests comparing responses from early and late participants also indicated no significant differences, reinforcing the reliability of the survey results.

Subsequent to SEM analysis, factor scores derived from the latent variable model were normalized to a 1–5 scale for NCA utilizing R software. A sample size of 10,000, conforming to suggested standards, was utilized to investigate the necessary effect size and statistical significance, determining the extent to which a variable or construct is a necessary condition for an outcome (Richter et al., 2020). Additionally, the analysis incorporated the CR-FDH (Conditional Efficiency - Restricted Disposal Hull) method, an NCA technique that discerns the minimum requisite level of a variable, based on the dataset’s efficiency frontier, necessary for achieving or surpassing a specific result. CR-FDH posits that data points are not permissibly disposed below this frontier, establishing that even a singular data point at a particular variable level substantiates its necessity for the result. This approach offers a stringent avenue for identifying and validating a variable as a necessary condition for an outcome (Dul, 2016).

## 4 Results

### 4.1 Measurement model

The internal consistency of each latent variable was evaluated using Cronbach’s alpha, while Composite Reliability (CR) values and factor loadings were employed to assess the convergent validity of the measurement model. As presented in Table 3, all constructs exhibited Cronbach’s alpha values ranging from 0.756 to 0.870, well above the recommended threshold of 0.7. Additionally, Average Variance Extracted (AVE) values ranged from 0.632 to 0.825, exceeding the standard minimum of 0.6, and factor loadings varied between 0.744 and 0.929, all surpassing the 0.7 benchmark. These indicators collectively suggest robust internal consistency and convergent validity (Hair et al., 2010). Furthermore, the discriminant validity of the model was established, as evidenced by the square roots of the AVEs for each latent variable, which were significantly greater than their respective inter-variable structural correlation coefficients. Each variable also demonstrated maximum loading on its intended latent construct as shown in Table 4, confirming the validity and effectiveness of the theoretical model (Chin, 1998).

**Table 3:** Factor loadings, composite reliability, and internal consistency.

Variables*	Factor loading	AVE	CR	Cronbach’s alpha
IT	0.798 ~ 0.901	0.697	0.873	0.793
OC	0.763 ~ 0.887	0.683	0.866	0.765
PP	0.862 ~ 0.913	0.791	0.919	0.804
RR	0.744 ~ 0.856	0.632	0.873	0.871
CD	0.832 ~ 0.891	0.753	0.901	0.77
AC	0.760 ~ 0.908	0.699	0.874	0.831
PBV	0.827 ~ 0.925	0.751	0.901	0.699
EP	0.865 ~ 0.929	0.818	0.931	0.801
CI	0.870 ~ 0.941	0.825	0.934	0.782

*IT, individual traits; OC, organizational climate; PP, project pressures; RR, rules and regulations; CD, cultural differences; AC, attitude toward compliance; PBV, perceived behavior value; EP, emotional positivity; CI, compliance intention.

**Table 4:** Result of discriminant validity.

	IT*	OC	PP	RR	CD	AC	PBV	EP	CI
IT	**0.835**^a^								
OC	0.458	**0.826**							
PP	0.235	0.453	**0.889**						
RR	0.19	0.231	0.321	**0.795**					
CD	0.389	0.294	0.203	0.28	**0.868**				
AC	0.083	0.217	0.198	0.312	0.325	**0.836**			
PBV	0.273	0.145	0.123	0.179	0.256	0.672	**0.867**		
EP	0.152	0.118	0.134	0.401	0.553	0.551	0.383	**0.904**	
CI	0.452	0.324	0.348	0.213	0.189	0.402	0.499	0.537	**0.908**

*IT, individual traits; OC, organizational climate; PP, project pressures; RR, rules and regulations; CD, cultural differences; AC, attitude toward compliance; PBV, perceived behavior value; EP, emotional positivity; CI, compliance intention.

a The bold values are the square root of AVE.

### 4.2 Structural model and hypothesis testing

This study validated 13 out of 15 proposed hypotheses (Table 5). Specifically, Hypothesis 10 (coefficient: 0.109) and Hypothesis 11 (coefficient: 0.082) were not supported, indicating that rules and regulations did not significantly impact employees’ perceived behavior value. Cultural differences did not have a significant effect on employees’ perceived behavior value. Hypothesis 1 (coefficient: 0.265, *p* < 0.001), Hypothesis 2 (coefficient: 0.297, *p* < 0.001), Hypothesis 3 (coefficient: 0.321, *p* < 0.001), Hypothesis 4 (coefficient: 0.338, *p* < 0.001), Hypothesis 5 (coefficient: 0.175, *p* < 0.01) and Hypothesis 6 (coefficient: 0.236, *p* < 0.001) were significantly supported, showing that individual traits and organizational climate had a substantial influence on attitude toward compliance, perceived behavior value, and emotional positivity. When the organizational climate increasingly views compliance as a pivotal job prerequisite, employees’ attitudes and affective responses toward compliance are anticipated to undergo enhancement. The presence of a greater number of factors within an individual’s traits that facilitate compliance-related endeavors is associated with a more optimal state of the organism factors. Hypothesis 7 (coefficient: 0.161, *p* < 0.05), Hypothesis 8 (coefficient: 0.181, *p* < 0.01), Hypothesis 9 (coefficient: 0.120, *p* < 0.05), and Hypothesis 12 (coefficient: 0.137, *p* < 0.05) were supported, indicating that project pressures impacted perceived behavior value and emotional positivity, rules and regulations had a certain influence on attitude toward compliance, and cultural differences influenced emotional positivity. The increased project pressure faced by employees may render it more difficult for them to maintain a high level of perceived behavioral value and emotional positivity. Furthermore, significant cultural differences can pose considerable challenges to the enhancement of employees’ emotional positivity. Finally, Hypothesis 13 (coefficient: 0.325, *p* < 0.001), Hypothesis 14 (coefficient: 0.288, *p* < 0.001), and Hypothesis 15 (coefficient: 0.368, *p* < 0.001) were validated, illustrating that attitude toward compliance, perceived behavior value, and emotional positivity significantly influenced compliance intention.

**Table 5:** Results of hypothesis testing.

			Standard		
Hypothesis	Path	Coefficient	Deviation	*t*-value	Conclusion
1	IT → AC	0.265***	0.063	4.832	Support
2	IT → PBV	0.297***	0.052	6.871	Support
3	IT → EP	0.321***	0.059	7.713	Support
4	OC → AC	0.338***	0.051	6.872	Support
5	OC → PBV	0.175**	0.053	2.832	Support
6	OC → EP	0.236***	0.052	4.282	Support
7	PP → PBV	0.161*	0.064	2.126	Support
8	PP → EP	0.181**	0.061	2.957	Support
9	RR → AC	0.120*	0.06	2.353	Support
10	RR → PBV	0.109	0.064	1.571	Unsupport
11	CD → PBV	0.082	0.065	1.332	Unsupport
12	CD → EP	0.137*	0.059	2.261	Support
13	AC → CI	0.325***	0.065	6.917	Support
14	PBV → CI	0.288***	0.051	7.281	Support
15	EP → CI	0.368***	0.063	9.738	Support

***p < 0.001; **p < 0.01; *p < 0.05.

### 4.3 Results of NCA

**Figure 2:**
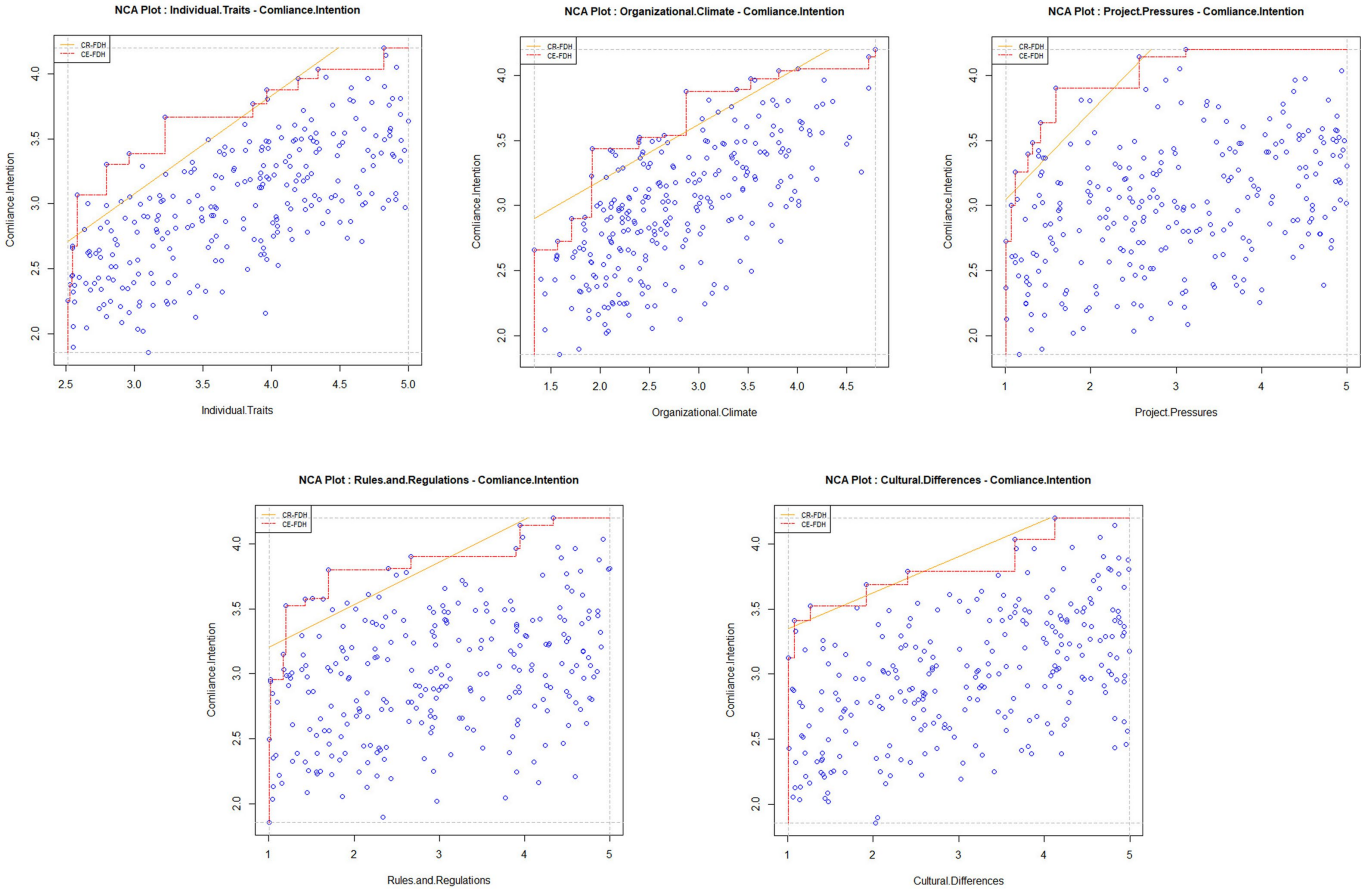
Scatterplots of all correlations.

The findings presented in Table 6 delineated that individual traits and organizational climate as robustly necessary for compliance intention, with precision levels of 95.2 and 91.9%, upper boundaries exceeding 5.8 and 8.1, and substantial effect sizes of 0.253 and 0.241, complemented by *p*-values well under 0.001. While project pressures, rules and regulations, and cultural differences are also determined as necessary to an extent, their comparatively lower effect sizes and varied levels of statistical significance indicated a relatively weaker necessity in influencing compliance intention. Figure 2 provides scatterplot visualizations for the entire spectrum of correlations.

**Table 6:** Results of NCA.

Condition	Precision	Upper bound	Range	Effect size (d)	*p*-value
Individual traits	95.20%	1.474	5.836	0.253	0.000
Organizational climate	91.90%	1.951	8.109	0.241	0.000
Project pressures	95.60%	0.986	9.373	0.105	0.167
Rules and regulations	95.60%	1.522	9.386	0.162	0.013
Cultural differences	98.50%	1.308	9.354	0.14	0.058

The condition refers to the calibrated fuzzy membership value; An effect size (d) between 0 and 0.1 is considered low; The permutation test in NCA analysis was conducted with 1,000 resamples.

Bottleneck analysis elucidated that with rising compliance intention levels, the bottleneck incidence for each condition increased correspondingly. At full compliance intention, individual traits and organizational climate attained bottleneck incidences of 79.5 and 86.7%, underscoring their substantial role. Concurrently, project pressures, rules and regulations, and cultural differences manifested bottleneck percentages of 42.7, 76.1, and 76.8%, reflecting their significance as necessary conditions, albeit with a reduced impact (Table 7). Collectively, these outcomes confirm individual traits and organizational climate as critical for high compliance intention, whereas the necessity of project pressures, rules and regulations, and cultural differences is more pronounced at elevated levels of intention, albeit to a lesser extent.

**Table 7:** Results of bottleneck analysis.

Compliance intention	Individual traits	Organizational climate	Project pressures	Rules and regulations	Cultural differences
0	NN*	NN	NN	NN	NN
10	NN	NN	NN	NN	NN
20	NN	NN	NN	NN	NN
30	NN	NN	NN	NN	NN
40	4.5	NN	NN	NN	NN
50	17	8.6	NN	NN	NN
60	29.5	24.2	8.1	4.7	NN
70	42	39.8	16.7	22.5	13.5
80	54.5	55.5	25.4	40.4	34.6
90	67	71.1	34	58.2	55.7
100	79.5	86.7	42.7	76.1	76.8

*NN, not necessary.

## 5 Discussion

### 5.1 Result analysis

**Figure 3:**
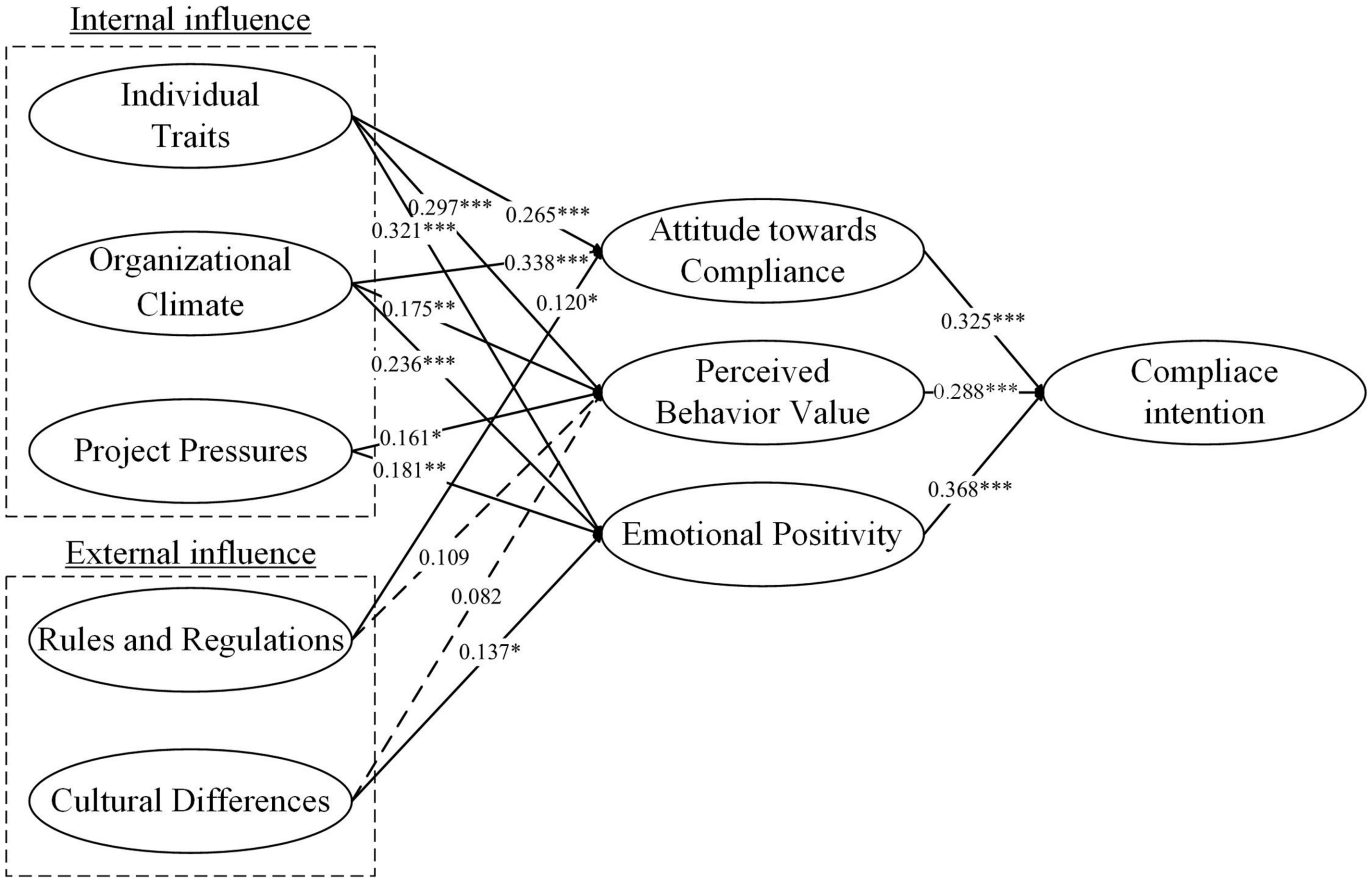
Hypothesis test results.

This study aims to explore the mechanism of environmental stimuli on the compliance psychology and behavior of international construction employees. The goal is to assist international contractors in identifying and prioritizing the factors most crucial for enhancing worker compliance. According to the results of SEM (Figure 3, ***p < 0.001, **p < 0.01, *p < 0.05, dashed lines indicate insignificant paths), five environmental factors have an impact on the compliance intentions of international construction employees by stimulating the organism. Compared to external organizational factors, internal factors have a greater impact on the organism. Notably, individual traits and organizational climate significantly affect three organism factors: attitude toward compliance, perceived behavior value, and emotional positivity. These findings support the perspectives of Lee and Foo (2022), Grojean et al. (2004), and Jeung and Chang (2021), which suggested that individual traits and organizational atmosphere can influence employees’ attitudes, emotions, and values.

Simultaneously, external factors such as rules and regulations and cultural differences have limited impact on the organism factors. In particular, the hypothesis regarding the influence of rules and regulations or cultural differences on employees’ perceived value was rejected, differing from the conclusions of Seegebarth on how cultural differences affect individual perceived values (Seegebarth et al., 2016). Furthermore, the NCA results revealed a strong necessity for individual traits and organizational climate, especially when the intention for compliance is high, indicating their critical importance to compliance intentions. In contrast, project pressure, rules and regulations, and cultural differences are also necessary conditions to some extent, but their necessity is relatively lower, especially when the level of compliance intention is not particularly high. This still supports Liu et al.’s research that project pressure will weaken employees’ compliance attitude, while supervision can enhance their compliance attitude (Liu et al., 2022).

International construction projects are characterized by their temporality and insularity. Employees often face significant feelings of unfamiliarity, cultural differences, and adaptation issues when moving from their home countries to host countries of the projects (Konanahalli et al., 2012). Their work and life are generally confined to the project site, forming a relatively closed organizational micro-ecosystem. In such contexts, employees’ psychology and behavior are mainly influenced by themselves, as well as colleagues and the organization. For instance, individuals with a strong sense of rules exhibit better attitudes toward compliance, and organizations that uphold a culture of compliance are less likely to experience violations internally (Langevoort, 2017). Additionally, in the micro-ecosystem of project organizations, employees generally do not have direct contact with the external environment, and most external information does not directly impact them. Legal regulations and cultural differences, among other information, are often filtered and absorbed by project organizations to influence employees’ behavioral decisions, such as through the issuance of worker behavior guidelines and the conducting of compliance training at the project level (McIlwraith, 2021). Therefore, individual traits and organizational climate can be considered key environmental stimuli in the compliance management of international construction projects.

### 5.2 Theoretical implications

This study explores the impact of environmental factors on the compliance intentions of international construction employees by applying and extending the SOR model. The research adapts the SOR model to the novel context of international construction projects and broadens its scope to encompass diverse environmental stimuli, including individual traits, organizational climate, project pressure, rules and regulations, and cultural differences. These stimuli are analyzed for their effects on key organism components such as attitudes toward compliance, perceived behavior value, and emotional positivity. Such an application and expansion of the SOR model offers fresh perspectives on its relevance and efficacy in multicultural and international business settings.

While previous studies have examined the impacts of organizational atmosphere and individual factors on compliance management (Neal et al., 2000; Zubaidah et al., 2012), they have predominantly done so from an organizational management perspective, often neglecting the psychological aspects of employees (Jaafar and Ajis, 2013). This research uncovers the distinct effects of both internal and external environmental factors on compliance intentions, offering an in-depth analysis of how these factors influence worker compliance behaviors in international construction projects. Importantly, the study highlights the significant influence of individual traits and organizational climate on the psychological and behavioral aspects of worker compliance. This revelation paves the way for novel compliance management strategies in international construction projects, emphasizing the critical role of employees’ psychological states.

Moreover, this study accentuates the distinctive micro-ecological characteristics of international construction projects, notably their temporality and insularity, and examines their influence on worker psychology and behavior. This approach offers fresh insights into the behavioral dynamics of employees within cross-cultural and evolving environments. The study also integrates the Necessary Condition Analysis (NCA) method to quantitatively evaluate the relative significance of different environmental factors in shaping compliance intentions in international construction projects. This integration presents an innovative analytical framework for investigating the complex interplay of various factors.

In summary, the theoretical contribution of this paper lies in applying and extending the SOR model to the field of international construction projects, providing an in-depth analysis of the impact of various environmental factors on compliance intentions, and offering new understandings and management strategies for international project management. This work lays a rich theoretical foundation and analytical tools for future research in this area.

### 5.3 Managerial implications

**Figure 4:**
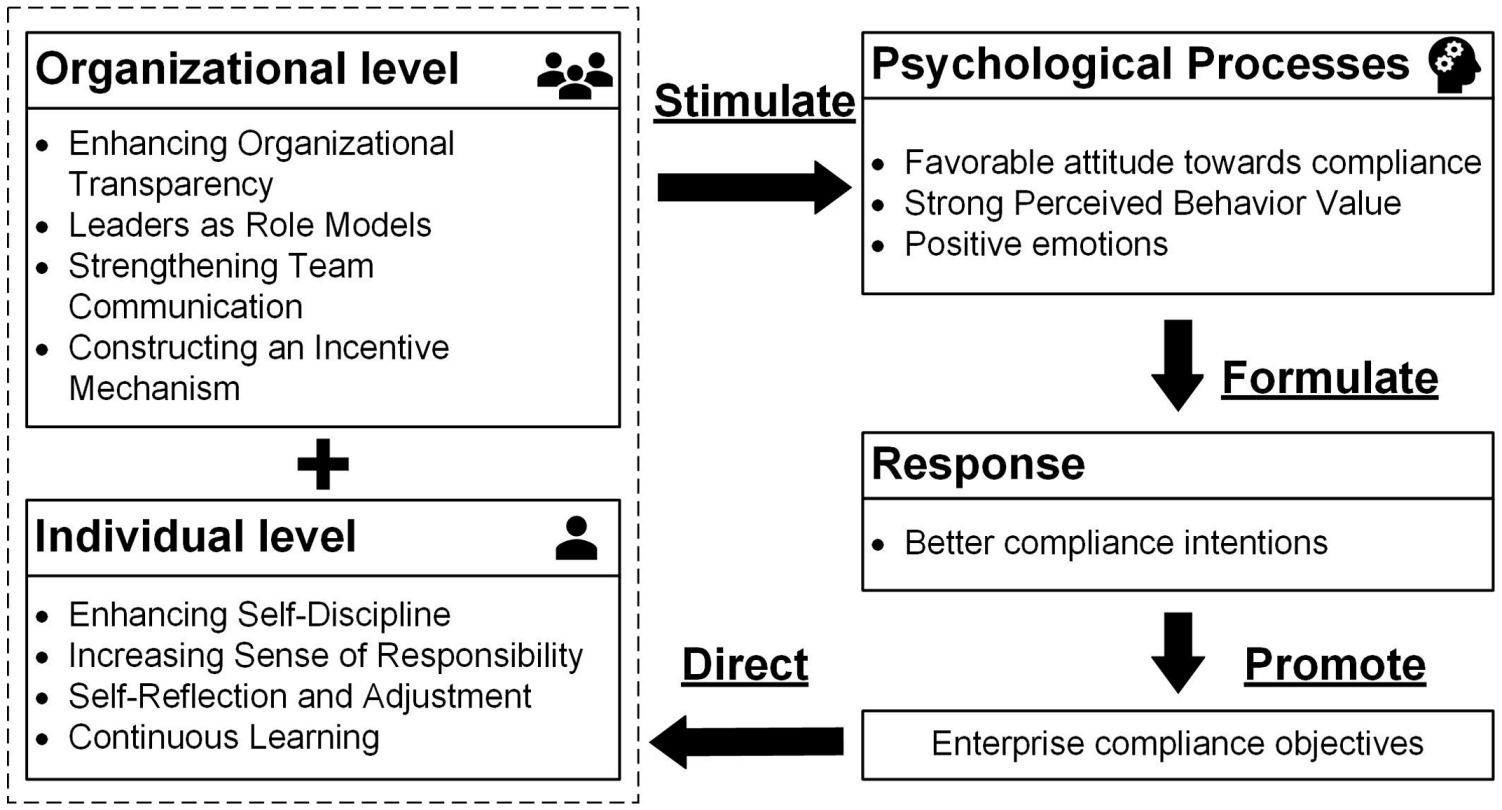
Dual compliance management strategies.

In managing compliance within international construction projects, objective factors such as rules and regulations, cultural differences, and project pressures invariably exist (Scott et al., 2011). While these environmental stimuli do impact the organism, businesses are largely unable to directly change these external factors. In contrast, aspects at the individual and organizational levels are key environmental stimuli that enterprises can more effectively manipulate. In response, this study proposes a binary compliance management strategy, as detailed in Figure 4, that concentrates on both the organizational structure and individual employees. This strategy enables international contractors to enact a series of targeted measures at both the organizational and personal levels. These measures are designed to positively influence employees’ psychological orientations, promoting attitudes supportive of compliance, enhancing value perception, and cultivating positive emotions. The aim is to develop strong compliance intentions among employees.

At the organizational level, it is imperative for international contractors to foster organizational transparency, a move that has been shown to reinforce worker trust (Berggren and Bernshteyn, 2007; Rawlins, 2008). Enhancing transparency not only fortifies team trust but also elevates the perceived importance of compliance among employees, thus encouraging their voluntary adherence to regulations. Leadership plays a critical role in this context, with leaders expected to exemplify moral standards, thereby setting a tone of integrity and compliance within the team (Sims and Brinkman, 2002). Additionally, it is essential for organizations to cultivate a communicative environment that supports the exchange of compliance-related knowledge and ideas (Jiacheng et al., 2010). This approach aids in developing a shared understanding of compliance issues and aligning viewpoints across the team. Furthermore, the implementation of well-designed incentive mechanisms is recommended. Such mechanisms should reward compliant behaviors, reinforcing positive actions, while also providing deterrents for non-compliance (Li and Hoffman, 2023). This balanced approach of rewards and penalties is pivotal in promoting and maintaining a culture of compliance within the organization.

At the individual level, enhancing self-discipline is crucial for employees. Highly self-disciplined individuals demonstrate clear judgment and rule adherence, especially when confronted with temptations to engage in non-compliant behaviors (Arthur and Graziano, 1996). This quality is especially vital for frontline project staff who regularly face a multitude of compliance challenges. Furthermore, elevating a sense of responsibility is imperative. According to Ali and Ahmad (2014), employees with heightened responsibility are more considerate of stakeholder needs and actively safeguard the company’s image and interests, often displaying greater positivity toward compliance initiatives. Cultivating self-reflection and the ability to adjust behavior in response to potential rule violations is another key trait. This self-awareness fosters a compliance-friendly mindset and enhances sensitivity to rule boundaries, which is instrumental in long-term adherence to correct procedures (Gerace et al., 2017). Additionally, fostering a culture of continuous learning is essential in an environment where rules and requirements are constantly evolving. This ongoing learning process enables employees to stay abreast of the latest compliance standards and skills. Engaging in reflective analysis of compliance issues across various scenarios deepens their understanding of the significance of compliance, thus acknowledging its role in bolstering the organization’s reputation and sustainable growth.

The binary compliance management strategy emphasized in this research underscores the critical role of frontline employees in a bottom-up approach to compliance management. This strategy, focusing on worker engagement in decision-making and problem-solving, contrasts with traditional top-down methods (Given, 1949; Lupton, 1991), facilitating the achievement of compliance objectives. Moreover, corporate compliance goals are not static; they evolve with increasing external regulatory demands, leading to more detailed and specific objectives. This dynamic nature of compliance goals guides adjustments in strategies at both organizational and individual levels, ensuring adaptability to changing external conditions.

### 5.4 Limitations and future research

This study, while insightful, has certain limitations. The snowball sampling method, primarily involving Chinese contractors, might limit the generalizability of the findings. Future research should focus on expanding the sample size and including contractors from diverse nationalities to enhance the applicability and precision of the results. Additionally, exploring a wider range of international engineering issues and integrating more varied methodological approaches could provide a broader perspective and deeper insights into global engineering challenges.

## 6 Conclusion

This study in international construction projects underscores the critical role of psychological and behavioral dynamics in worker compliance, heavily influenced by environmental stimuli. The research demonstrates that internal organizational factors, notably Individual Traits and Organizational Climate, are more influential on compliance intentions than external ones. The introduced ‘Binary Micro-Ecological Compliance Management Strategy’ champions a bottom-up approach, placing employees at the forefront of compliance activities. This strategy not only provides a practical guide for enhancing compliance management but also enriches the theoretical framework by elucidating the impact of environmental stimuli on worker behavior, laying the groundwork for future research in this area.

## Data availability statement

The original contributions presented in the study are included in the article/supplementary material, further inquiries can be directed to the corresponding author.

## Ethics statement

The studies involving humans were approved by Ethics Committee of School of Civil Engineering, Southeast University. The studies were conducted in accordance with the local legislation and institutional requirements. The participants provided their written informed consent to participate in this study.

## Author contributions

TC: Conceptualization, Data curation, Funding acquisition, Investigation, Methodology, Resources, Software, Supervision, Writing – original draft, Writing – review & editing. YW: Conceptualization, Formal analysis, Methodology, Project administration, Supervision, Writing – original draft, Writing – review & editing. XD: Conceptualization, Resources, Writing – review & editing. XW: Software, Visualization, Writing – review & editing. YY: Visualization, Writing – review & editing.
